# The Effect of Multidisciplinary Team Discussion Intervention on the Prognosis of Advanced Colorectal Cancer

**DOI:** 10.7150/jca.56171

**Published:** 2021-04-07

**Authors:** Huaqi Zhang, Jishang Yu, Zhewei Wei, Wenhui Wu, Changhua Zhang, Yulong He

**Affiliations:** 1 Digestive Disease Center, Seventh Affiliated Hospital of Sun Yat-Sen University.; 2 Gastrointestinal Surgery Center, First Affiliated Hospital of Sun Yat-Sen University.

**Keywords:** multidisciplinary team, clinical decision-making, colorectal neoplasms, prognosis, treatment efficiency

## Abstract

**Purpose:** The effects of multidisciplinary team discussion intervention on the treatment and prognosis of advanced colorectal cancer are still controversial. Large sample size studies to evaluate the efficacy in patients with advanced colorectal cancer are lacking.

**Materials and Methods:** We statistically analyzed the data of surgical patients diagnosed with advanced colorectal cancer from 2008 to 2014 by retrospective analysis. Patients were divided into two groups according to whether or not they received multidisciplinary team discussion intervention. After at least 3 years of follow up, differences between two groups were compared with respect to treatment process and patient prognosis.

**Results:** The time to treatment in intervention group was shorter (9.6 ± 4.2 days vs 10.7 ± 5.6 days; p= 0.002). There were no significant differences in recurrence and metastasis rate between the two groups. Multivariate survival analysis suggested that multidisciplinary team discussion intervention reduced the risk of death (HR = 0.677; p = 0.006). And it had significant interaction with tumor invasion and tumor stage, and especially had beneficial effects in the tumor stage IV subgroup (p=0.005) and tumor invasion T4 subgroup (p<0.001).

**Conclusion:** Multidisciplinary team discussion intervention accelerated the treatment process and reduced the death risk of patients with advanced colorectal cancer, especially improved the overall survival of stage IV and invasion T4 patients. The clinical characteristics of tumor invasion and tumor stage must be the primary considerations when judging whether patients need to conduct multidisciplinary team discussions.

## Introduction

Colorectal cancer (CRC) is a disease with high morbidity and mortality 1. With improved understanding of the disease along with development of medical technology, there is constant improvement and diversification of diagnosis and treatment options for CRC 2, 3. Although some accurate and effective therapeutic regimens are available for early stage CRC 4, there are no definitive or unified treatment plans for CRC with lymph node, local, or distant metastasis 2, 3. In patients with advanced CRC, clinicians are often unable to accurately judge patient condition upon diagnosis because of the complexity of the disease. Clinicians typically make treatment decisions according to clinical experience and existing treatment guidelines. The multidisciplinary team (MDT) discussion model was developed in order to solve this problem in many countries 5, 6. MDT is a clinical treatment model that facilitates multidisciplinary discussion during the process of disease diagnosis and treatment 7. Regular collaborative discussion of experts in many disease-related disciplines allows for improved accuracy of CRC diagnoses and also provides an avenue to make suggestions during the treatment process 8. MDT facilitates professional discussion and evaluation for many aspects of the clinical decision-making process for malignant disease, such as disease diagnosis, preoperative evaluation, preoperative treatment, operation scheme, and postoperative treatment 9, 10. Multiple disciplines participate in the patient treatment process to accurately judge the patient's condition and formulate a treatment plan in line with individual patient characteristics, such that patients are able to achieve a better prognosis.

MDT has been used in the treatment of CRC, and studies have shown that the MDT model benefits CRC patients for many reasons. First, MDT speeds up the treatment process for patients with CRC. The use of MDT intervention during clinical diagnosis and treatment improves the efficiency of initial disease assessment across several departments, shortens the time from definitive diagnosis to first treatment, and increases the overall efficiency of the patient treatment process 11. However, some studies have shown that the introduction of MDT prolongs the time for patients to receive treatment, although this remains controversial 12. Second, MDT intervention influences physician choice of therapeutic schedule. The results of some studies show that after MDT, 13-29% of patients changed their original treatment plan, including their adjuvant treatment plan and operation plan; in these cases, the main factors affecting the choice of treatment plan were the re-evaluation of imaging results and tumor conditions 7, 13, 14. Finally, MDT improves the prognosis of CRC patients and also increases the overall survival and progression-free survival of patients. For patient cases that underwent MDT review and according treatments, survival rate was significantly improved 15, 16. Furthermore, a study by Chien-Hsin Chen also confirmed that MDT improves 3-year survival of CRC patients with hepatic metastasis or pulmonary metastasis 17.

However, there is a lack of clinical data with a large sample size to evaluate the overall effect of MDT intervention in patients with advanced CRC. Moreover, there is no unified conclusion on the efficiency of MDT or the effect of MDT on the prognosis of patients with advanced CRC. Thus, the purpose of this study was to retrospectively analyze clinical data from patients with advanced CRC with or without MDT intervention to further clarify the effects of MDT on the treatment and prognosis of advanced CRC.

## Materials and Methods

### Clinical Data

We retrospectively analyzed clinical data from patients with advanced CRC (stage III and stage IV, according to AJCC/TNM staging 8th edition). All patients were newly diagnosed between January 2008 and December 2014 at the same hospital. The patients received surgical treatment and perioperative treatment by the same medical team at the same hospital where they were diagnosed. All patients must be followed up for at least 3 years and had complete follow-up records. We excluded patients with extensive systemic metastasis that could not be treated surgically, patients with malignant tumors in other organs, patients who received postoperative adjuvant therapy in other hospitals, and patients who underwent emergency surgery. Patients were divided into an MDT group and a non-MDT group; patients in the MDT group chose a treatment plan according to the results of an MDT discussion, whereas the treatment plan for patients in the non-MDT group was determined by a physician according to treatment guidelines and clinical treatment experience. The demographic data and clinical indexes, including gender, age, tumor location, tumor pathological classification, differentiation degree, depth of invasion, tumor histological stage (according to AJCC/TNM staging 8th edition) and serum tumor markers carcinoembryonic antigen (CEA) and carbohydrate antigen 19-9 (CA 19-9) were analyzed. In addition, we statistically analyzed treatment-related indicators such as period between the dates of hospital admission and having surgery, mode of operation and number of regional lymph nodes metastasis to determine the effect of MDT on patient treatment. Ethical committees of the Seventh Affiliated Hospital of Sun Yat-sen University approved this study and approved the application for exemption from informed consent (KY-2020-024-01). The whole study complied with the requirements of the Declaration of Helsinki.

### Multidisciplinary Team Discussion Intervention

We held MDT discussions regularly each Monday since 2012. All patients who were initially diagnosed with clinical stage III or stage IV CRC would have MDT discussion immediately following the diagnosis, and MDT would be continued until the patients completed the entire treatment process. Patients were informed before MDT discussions, and they had right to refuse to accept MDT discussion. The MDT discussion provided decision-making suggestions on patient condition, preoperative treatment, operation scheme, and postoperative adjuvant treatment. The MDT discussion was led by the attending physician of the surgical treatment department; the imaging department, the oncology department, the anesthesiology department, the operating room, the radiotherapy department, and other disease-related departments participated in the discussion. If necessary, the liver surgery department, the thoracic surgery department, and other relevant departments were also invited to participate in the discussion. After the discussion, the attending physicians in the surgical treatment department would summarize the results of the discussion. These attending physicians were responsible for implementing the relevant treatment plan in a manner that strictly adhered to the results of the MDT discussion.

### Follow up

Patient follow ups were scheduled every 3 months during the first year, every 6 months during the second year, and once per year thereafter. The patients were asked to have regular examinations of serum tumor markers, chest and abdominal enhanced CTs, pelvic MRIs, endoscopies, and other related examinations. This study included follow up data through December 2017.

### Statistical analysis

Rank sum test was used for rank data, chi-square test was used for fixed type variables, and the t-test was used for statistical analysis of quantitative continuous variables. The Kaplan-Meier method and log-rank test were used to compare overall survival between two groups. Hazard ratio (HR) and 95% CI for HR were used to evaluate the effect of MDT intervention on the risk of death by Cox proportional hazard (PH) model analyses, with all variables conforming to PH assumption. To more accurately evaluate the effect of MDT intervention on the overall survival of patients, we further adopted multivariate Cox proportional hazard model analyses to adjust for existing and potential confounding factors. We established adjusted models to evaluate the effect strength of covariates. The adjustment order was determined according to the influence degree of the confounding factors on HR, which also reflected the different influence degrees of different confounding factors on the results. The uncorrected model did not adjust for confounding factors; the basic adjusted model adjusted the age variable and gender variable; the adjusted model 1 was additionally adjusted the tumor invasion variable; the adjusted model 2 was additionally adjusted tumor invasion, tumor stage, pathological type, and differentiation degree; and the adjusted model 3 was additionally adjusted all possible variables, including tumor invasion, tumor stage, pathological type, differentiation degree, tumor location and radical resection. We next performed subgroup analyses to estimate the correlation between MDT intervention and overall survival of all advanced CRC patients by covariates, including age, gender, tumor invasion, tumor stage, pathological type, differentiation degree, tumor location and radical resection. P for interaction of binary variables were tested by Cox regression model, p for interaction of polytomous variables were tested by likelihood-ratio test. Subgroup analyses were performed using the adjusted model 3. An inspection level α = 0.05, p < 0.05 was defined as statistically significant. SPSS 23.0 (IBM, Armonk, NY, USA) was used for statistical analysis of data.

## Results

### Demographic data and baseline clinical characteristics of patients

According to the study criteria, a total of 879 patients with CRC were included in this study; 473 patients were in the MDT group and 406 patients were in the non-MDT group. Ten patients were lost during the follow up period; the total follow up rate was 98.9%, the average follow up time was 43.8 ± 29.5 months, and the median follow up time was 43.8 months. Since the proportion of variables with theoretical frequency less than five is more than 20%, to meet the application conditions of the chi-square test we combined tumor invasion depth T1 and T2 into the 'T1+T2' variable. Furthermore, for degree of differentiation, we merged 'high differentiated' variable into the 'others' variable. Between the two groups, there were no significant differences in sex, age distribution, tumor location, pathological type, differentiation degree, tumor stage or tumor marker (CA 19-9) (Table 1). In terms of the degree of invasion, the proportion of stage T4 in the MDT group was higher than that in the non-MDT group (80.5% vs 12.8%), whereas the proportion of stage T3 was higher in the non-MDT group (78.3% vs 14.0%; p < 0.001). More patients in the non-MDT group had a CEA level > 5 ng/ml (35.3% vs. 43.1%; p = 0.018).

### Effect of MDT on time to treatment and surgery quality

The time to treatment (TTT) was the period between the dates of hospital admission and having initial treatment. The average TTT for the MDT group was shorter than that for the non-MDT group (9.6 ± 4.2 days vs 10.7 ± 5.6 days, p=0.002). There were no significant differences between the two groups for the proportion of patients who underwent radical surgery or the proportion of patients with rectal cancer who underwent total mesorectal excision (TME). There were also no significant differences between the two groups with respect to the proportion of patients with ≥ 12 regional lymph nodes (RLN; Table 2).

### Effect of MDT on prognosis of CRC patients

**Figure 1:**
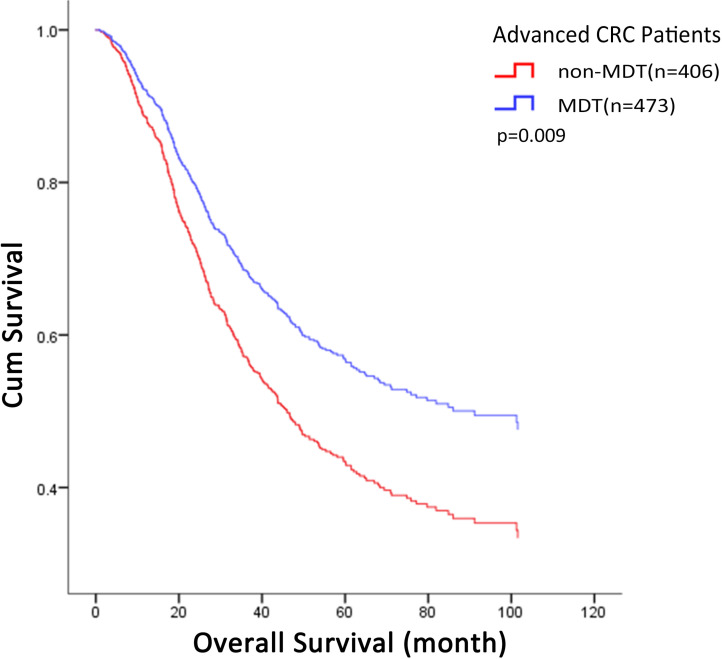
MDT-related multivariate COX proportional hazard model analyses. The overall cumulative survival rate in MDT group was higher than that in non-MDT group (p=0.009). MDT: multidisciplinary team.

Three years after operation, there were no significant differences in recurrence or metastasis rates between the two groups (MDT: 50.32% vs non-MDT: 48.03%; p = 0.499). Due to gradual improvement of the department and MDT system, a higher proportion of T4 patients with poorer prognosis received MDT discussion intervention, which influenced Kaplan-Meier survival curves. The overall survival was analyzed using the Kaplan-Meier method and log-rank test. The results showed that the median overall survival was 53.7 months for the MDT group and 68.4 months for the non-MDT group; the 3-year survival rate was 61.1% for the MDT group and 60.8% for the non-MDT group. There was no significant difference in overall survival between the two groups (p = 0.374). Univariate Cox proportional hazard model analysis indicated that MDT discussion intervention had no significant effect on the death risk of CRC patients (p = 0.375). Univariate Cox regression analysis could not eliminate interference of confounding factors, and therefore, we established adjusted models to further clarify confounding factors that may influence the efficacy of MDT. Controlling for variables of tumor invasion, tumor stage, differentiation degree, and pathological type further highlighted the protective effect of MDT, suggesting that the above factors are important and related to the therapeutic effect of MDT (Table 3). Fully adjusted multivariate Cox proportional hazard model analyses suggested that MDT discussion intervention could be used as a protective factor to reduce the risk of death after controlling for all possible confounding factors (HR = 0.686; 95% CI = 0.518-0.910; p = 0.009; Table 4). After adjustment, the cumulative survival rate of patients in the MDT group was higher than that in the non-MDT group (Fig. 1).

**Figure 2:**
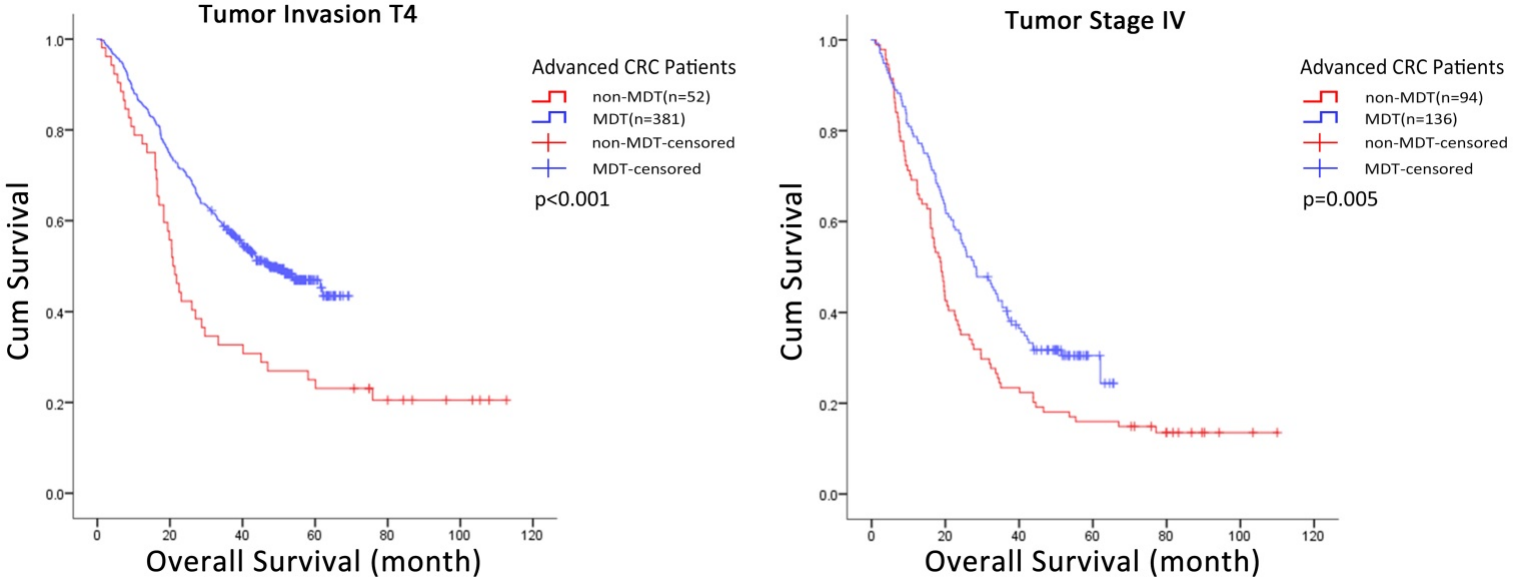
Kaplan-Meier curves of the overall survival of CRC patients in subgroup. MDT discussion intervention improved the accumulate survival rate of patients in T4 subgroup (p<0.001) and stage IV subgroup (p=0.005). MDT: multidisciplinary team; CRC: colorectal cancer.

We used subgroup Cox regression analysis to further evaluate the correlation between covariates and the effect of MDT discussion intervention on the risk of death in all advanced CRC patients. The results suggested that some covariates had no significant correlation with MDT discussion intervention including age (Δdf=1, Δχ^2^=0.317, p>0.05), gender (p = 0.455), tumor location (Δdf=1, Δχ^2^=0.277, p>0.05), degree of differentiation (Δdf=1, Δχ^2^=3.741, p>0.05), radical resection groups (p = 0.257) or pathological type (Δdf=1, Δχ^2^=0.752, p>0.05). In contrast, tumor invasion (Δdf=1, Δχ^2^=8.478, p<0.05), tumor stage (p = 0.005) did show significant correlation with MDT discussion intervention, and therefore, may affect the effect strength of MDT discussion intervention (Table 5). Further, the Kaplan-Meier method and Log-rank test was used to analyze these two groups of data with statistical differences, and the results showed that MDT discussion intervention had significant influence on the overall survival of T4 (p < 0.001) and stage IV (p = 0.005) CRC patients (Fig. 2, Table S1).

## Discussion

In this study, we used a retrospective method to compare clinical treatment and prognosis of a large sample of CRC patients who were treated with or without the use of MDT discussion intervention. There were no significant differences in demographic data or basic clinical indexes between the MDT and non-MDT groups, and had the same surgery quality of two groups. Moreover, there were no significant differences in prognosis-related indexes between the two groups, including overall survival rate, recurrence and metastasis rate. A univariate Cox regression analysis showed that MDT discussion intervention did not affect overall survival for all patients. However, upon eliminating the interference of confounding factors by multivariate Cox regression analysis, the protective effects of MDT intervention on overall survival became apparent. Further subgroup analyses indicated that tumor invasion, tumor stage were the main covariates affecting the efficacy of MDT. MDT discussion intervention had a protective effect on patients in the tumor stage IV subgroup and tumor invasion T4 subgroup.

The effect of MDT discussion intervention on the efficiency of the treatment process is a primary concern for many physicians, who fear that multiple MDT discussions may delay treatment for patients with CRC. However, MDT discussion intervention during the process of clinical diagnosis and treatment has been shown to improve treatment efficiency 18, increase the efficiency of multiple departments assessing patient condition before treatment, and shorten the time from definitive diagnosis to first treatment 11. The results of our study, which are consistent with the conclusions of other researchers, demonstrate that use of the MDT model throughout the entire process of CRC diagnosis and treatment shortens the duration of the time to initial treatment. There was no significant difference in the quality of operation between the two groups. However, due to the improvement of surgical technology and equipment, the quality of operation is not a main factor affecting the prognosis of patients.

The positive effect of MDT on the survival of patients with CRC remains controversial 19. Munro et al. reported that the survival rate of patients was significantly improved if they were diagnosed and treated after MDT discussion intervention (63.1% vs 48.2%), and MDT reduced the risk of death as an independent risk factor 16. Another previous study reported that 3-year overall survival rates in CRC and hepatic metastasis patients with or without MDT intervention were 48.75% and 24.21%, respectively 17. The results of our study show a similar trend as the published literature. Our study suggests that, as an independent protective factor, MDT can reduce the risk of death from colorectal cancer. The risk of death in patients after MDT discussion intervention is 0.677 times lower than that in patients who did not receive MDT discussion intervention. However, the overall survival of advanced CRC patients involves a variety of factors and the target population of MDT applications remains unclear. Yueh-Han Hsu et al. 20 reported that in CRC, the HR with MDT intervention was 0.93 in patients with stage III CRC and 0.88 in patients with stage IV CRC. MDT had less of an effect on the risk of death in patients with stage III CRC. However, the resection rate of liver metastases in patients with stage IV CRC increased from 19.6% to 35.2%, the resection rate of pulmonary metastases increased from 12.4% to 14.3%, and the 3-year survival rate of patients with stage IV CRC increased from 25.6% to 38.2% 21. The results of a study from van der Vlies et al. 22 show that perioperative MDT can reduce the incidence of postoperative complications in frail colorectal cancer patients with elderly age and many underlying diseases, but it cannot effectively prolong overall survival time. Moreover, another study has shown that adjuvant chemotherapy regimens and radical resection rates are risk factors that affect the risk of death after MDT 23. According to our subgroup analyses, the efficacy of MDT intervention was mainly affected by tumor invasion and tumor stage. MDT had a protective effect in the tumor stage IV subgroup and also in the tumor invasion T4 subgroup. This emphasizes the need to primarily consider the clinical characteristics of tumor invasion and tumor stage in the process of MDT.

There are some limitations to this study. First, we mainly analyzed the data through retrospective studies, which may have created a certain selective bias. Due to the different criteria for MDT discussion intervention, MDT priority is often given to patients with complex conditions, whereas progressive patients with more definitive conditions will often enter directly into treatment. This makes the MDT and non-MDT groups biased in the severity of disease. Therefore, in the process of statistical analysis, we used adjustive statistical methods to reduce the bias. Second, there is no standard discipline configuration for MDT in patients with a definitive condition, which may alter the effectiveness of MDT discussion intervention. Third, all patients in this study were admitted to the hospital for surgical treatment, but some patients were enrolled in the group after receiving neoadjuvant therapy. Thus, it was not possible to evaluate the effect of MDT discussion intervention on preoperative treatment or the effect of preoperative treatment on patient prognosis and overall survival. Finally, great strides have been made to improve efficacy of therapeutic drugs and technologies applied to clinical treatment. With respect to survival analysis, it was difficult to differentiate between the influence of MDT discussion intervention and the effectiveness of new postoperative chemotherapy on overall patient survival. But according to our database, all chemotherapy regimens were first-line chemotherapy regimens recommended in NCCN guidelines between 2008 and 2014, we think that the impact of the development of chemotherapy regimens on our research conclusions is acceptable.

In conclusion, MDT discussion intervention can accelerate the treatment process of advanced CRC. MDT discussion intervention can significantly reduce the risk of death of patients with advanced CRC, and especially improve the overall survival of stage IV and invasion T4 CRC patients. The clinical characteristics of tumor invasion and tumor stage must be the primary consideration when judging whether patients need to conduct MDT discussions. A unified and standardized implementation for MDT intervention remains to be established, and more prospective clinical studies with large sample sizes are needed to provide a theoretical basis for evaluating the effectiveness of MDT intervention.

## Supplementary Material

Supplementary table S1.

## Figures and Tables

**Table 1:** Demographic data and clinical characteristics

Variables	MDT (n = 473), n (%)	non-MDT (n=406), n (%)	p-value
**Gender**			0.139
Male	242 (51.2)	228 (56.2)	
Female	231 (48.8)	178 (43.8)	
**Age (years)**			0.169
<46	39 (8.2)	49 (12.1)	
46-60	209 (44.2)	171 (42.1)	
>60	225 (47.6)	186 (45.8)	
**Tumor Location**			0.420
Ascending Colon	99 (20.9)	91 (22.4)	
Transverse Colon	20 (4.2)	20 (4.9)	
Descending Colon	164 (34.7)	116 (28.6)	
Rectum	186 (39.3)	176 (43.3)	
Multi-source Tumor	4 (0.8)	3 (0.7)	
**Pathological Type**			0.068
Villous Adenocarcinoma	14 (3.0)	15 (3.7)	
Tubular Adenocarcinoma	386 (81.6)	341 (84.0)	
Mucous Adenocarcinoma	51 (10.8)	43 (10.6)	
Signet Ring Cell Carcinoma	10 (2.1)	6 (1.5)	
Others	12 (2.5)	1 (0.2)	
**Differentiation Degree**			0.433
Moderately	335 (70.8)	286 (70.4)	
Poorly	131 (27.7)	109 (26.8)	
Others	7 (1.5)	11 (2.7)	
**Tumor Invasion**			<0.001^a^
T1+T2	26 (5.5)	36 (8.9)	
T3	66 (14.0)	318 (78.3)	
T4	381 (80.5)	52 (12.8)	
**Tumor Stage**			0.060
III	337 (71.2)	312 (76.8)	
IV	136 (28.8)	94 (23.2)	
**Serum Tumor Markers**			
***CEA (ng/ml)***			
>5	167 (35.3)	175 (43.1)	0.018^a^
0-5	306 (64.7)	231 (56.9)	
***CA19-9 (U/ml)***			
>35	148 (31.3)	113 (27.8)	0.263
0-35	325 (68.7)	293 (72.2)	

CEA: carcinoembryonic antigen; CA19-9: carbohydrate antigen 19-9; a: p<0.05 as statistical significance.

**Table 2:** Quality of colorectal surgery

Variables	MDT (n = 473), n (%)	non-MDT (n=406), n (%)	p-value
**Radical Resection**			0.059
Radical Resection	400 (84.6)	361 (88.9)	
Not Radical Resection	73 (15.4)	45 (11.1)	
**RLN**			0.389
≥12	349 (73.8)	289 (71.2)	
<12	124 (26.2)	117 (28.8)	

MDT: multidisciplinary team; TME: total mesorectal excision; RLN: regional lymph nodes.

**Table 3:** Risk of overall survival for MDT intervention

Adjustment Model	HR	95%CI	p-value
Unadjusted Model	1.091	0.900-1.322	0.375
Basic Adjusted Model	1.117	0.921-1.354	0.263
Adjusted Model 1	0.697	0.527-0.924	0.012^a^
Adjusted Model 2	0.655	0.494-0.868	0.003^a^
Adjusted Model 3	0.686	0.518-0.910	0.009^a^

Unadjusted Model: not adjusted for confounding factors; Basic Adjusted Model: additionally adjusted the age variable and gender variable; Adjusted Model 1: additionally adjusted the age, gender, tumor invasion variable; Adjusted Model 2: additionally adjusted the age, gender, tumor invasion, tumor stage, pathological type, and differentiation degree; Adjusted Model 3: additionally adjusted all possible variables, including age, gender, tumor invasion, tumor stage, pathological type, differentiation degree, tumor location and radical resection; CI: confidence interval; HR: hazard ratio; MDT: multidisciplinary team. a: p<0.05 as statistical significance.

**Table 4:** Fully adjusted multivariate COX proportional hazard model analyses of overall survival

Variables	HR	95%CI	P-value
**Group**			
non-MDT	1.000		
MDT	0.686	0.518-0.910	0.009^a^
**Age (years)**			
<46	1.000		
46-60	0.878	0.630-1.224	0.444
>60	1.195	0.863-1.655	0.284
**Gender**			
Male	1.000		
Female	0.855	0.704-1.040	0.117
**Tumor Invasion**			
T1+T2	1.000		
T3	1.997	1.190-3.350	0.009^a^
T4	2.994	1.735-5.165	<0.001^a^
**Tumor Stage**			
Stage III	1.000		
Stage IV	1.780	1.401-2.261	<0.001^a^
**Degree of Differentiation**			
Moderately Differentiated	1.000		
Poorly Differentiated	1.541	1.242-1.912	<0.001^a^
Others	1.069	0.551-2.074	0.843
**Tumor Location**			
Ascending Colon	1.000		
Transverse Colon	0.912	0.549-1.515	0.722
Descending Colon	1.002	0.764-1.314	0.987
Rectum	1.361	1.056-1.753	0.017^a^
Multi-source Tumor	0.817	0.278-2.405	0.714
**Radical Resection**			
Not	1.000		
Yes	0.258	0.195-0.343	<0.001^a^
**Pathological Type**			
Villous Adenocarcinoma	1.000		
Tubular Adenocarcinoma	1.709	0.901-3.241	0.101
Mucous Adenocarcinoma	2.010	1.005-4.019	0.048^a^
Signet Ring Cell Carcinoma	4.633	1.957-10.970	<0.001^a^
Others	1.917	0.711-5.171	0.198

MDT: multidisciplinary team; HR: hazard ratio; CI: confidence interval. a: p<0.05 as statistical significance.

**Table 5:** HR of MDT on overall survival of different subgroups

Subgroup	HR	95%CI	p for Interaction
Age	1.031	0.772-1.378	>0.05
Gender	0.865	0.591-1.266	0.455
Tumor Invasion	0.591	0.395-0.884	<0.05^a)^
Tumor Stage	0.568	0.384-0.840	0.005^a)^
Degree of Differentiation	0.640	0.461-0.889	>0.05^a)^
Tumor Location	0.996	0.847-1.172	>0.05
Radical Resection	1.306	0.823-2.073	0.257
Pathological Type	0.715	0.525-0.975	>0.05^a)^

Subgroup analyses were performed with adjusted model 3; MDT: multidisciplinary team; HR: hazard ratio; CI: confidence interval. a: p<0.05 as statistical significance.

## Abbreviations

CRC: colorectal cancer
MDT: multidisciplinary team
CEA: carcinoembryonic antigen
CA 19-9: carbohydrate antigen 19-9
HR: hazard ratio
PH: proportional hazard
TTT: time to treatment
TME: total mesorectal excision
RLN: regional lymph nodes
CI: confidence interval
