# Supplementary material for: The Effect of Multidisciplinary Team Discussion Intervention on the Prognosis of Advanced Colorectal Cancer
Source: J Cancer. 2021 Apr 7;12(11):3307–14. doi: 10.7150/jca.56171 (PMC8100813; doi:10.7150/jca.56171)
Supplement: Supplementary file 1 — Supplementary table S1. [file jcav12p3307s1.pdf]

Table S1. Effect of MDT on overall survival of subgroups with statistical differences

Variables		P-value
Tumor Invasion	T1+T2	0.662
	T3	0.775
	T4	<0.001[a]
Tumor Stage	Stage III	0.085
	Stage IV	0.005[a]

Effect of MDT on overall survival was analyzed by Kaplan-Meier method and Log-rank test.

MDT: multidisciplinary team.

a: $p<0.05$ as statistical significance
